# Chicken antibodies against venom proteins of *Trimeresurus stejnegeri* in Taiwan

**DOI:** 10.1590/1678-9199-JVATITD-2020-0056

**Published:** 2020-11-20

**Authors:** Chi-Hsin Lee, Chia-I Liu, Sy-Jye Leu, Yu-Ching Lee, Jen-Ron Chiang, Liao-Chun Chiang, Yan-Chiao Mao, Bor-Yu Tsai, Ching-Sheng Hung, Chi-Ching Chen, Yi-Yuan Yang

**Affiliations:** 1 School of Medical Laboratory Science and Biotechnology, College of Medical Science and Technology, Taipei Medical University, Taipei, Taiwan.; 2 Graduate Program in Medical Biotechnology, College of Medical Science and Technology, Taipei Medical University, Taipei, Taiwan.; 3 Graduate Institute of Medical Sciences, College of Medicine, Taipei Medical University, Taipei, Taiwan.; 4 Department of Microbiology and Immunology, College of Medicine, Taipei Medical University, Taipei, Taiwan.; 5 The Center of Translational Medicine, Taipei Medical University, Taipei, Taiwan.; 6 Center for Research, Diagnostics and Vaccine Development, Centers for Disease Control, Ministry of Health and Welfare, Taiwan.; 7 College of Life Sciences, National Tsing Hua University, Hsinchu, Taiwan.; 8 Division of Clinical Toxicology, Department of Emergency Medicine, Taichung Veterans General Hospital, Taichung, Taiwan.; 9 Navi Bio-Therapeutics Inc., Taipei, Taiwan.; 10 Department of Laboratory Medicine, Wan Fang Hospital, Taipei Medical University, Taipei, Taiwan.; 11 Department of Pathology and Laboratory Medicine, Landseed Hospital, Taoyuan, Taiwan.; 12 Core Laboratory of Antibody Generation and Research, Taipei Medical University, Taipei, Taiwan.

**Keywords:** Trimeresurus stejnegeri, IgY antibody, Phage display technology, Single-chain variable fragment antibody

## Abstract

**Background:**

The venom of bamboo vipers (*Trimeresurus stejnegeri* - TS), commonly found in Taiwan, contains deadly hemotoxins that cause severe envenomation. Equine-derived antivenom is a specific treatment against snakebites, but its production costs are high and there are some inevitable side effects. The aim of the present work is to help in the development of an affordable and more endurable therapeutic strategy for snakebites.

**Methods:**

*T. stejnegeri* venom proteins were inactivated by glutaraldehyde in order to immunize hens for polyclonal immunoglobulin (IgY) antibodies production. After IgY binding assays, two antibody libraries were constructed expressing single-chain variable fragment (scFv) antibodies joined by the short or long linker for use in phage display antibody technology. Four rounds of biopanning were carried out. The selected scFv antibodies were then further tested for their binding activities and neutralization assays to TS proteins.

**Results:**

Purified IgY from egg yolk showed the specific binding ability to TS proteins. The dimensions of these two libraries contain 2.4 × 10^7^ and 6.8 × 10^7^ antibody clones, respectively. An increase in the titers of eluted phage indicated anti-TS clones remarkably enriched after 2^nd^ panning. The analysis based on the nucleotide sequences of selected scFv clones indicated that seven groups of short linkers and four groups of long linkers were identified. The recombinant scFvs showed significant reactivity to TS venom proteins and a cross-reaction to *Trimeresurus mucrosquamatus* venom proteins. In *in vivo* studies, the data demonstrated that anti-TS IgY provided 100% protective effects while combined scFvs augmented partial survival time of mice injected with a lethal amount of TS proteins.

**Conclusion:**

Chickens were excellent hosts for the production of neutralization antibodies at low cost. Phage display technology is available for generation of monoclonal antibodies against snake venom proteins. These antibodies could be applied in the development of diagnostic kits or as an alternative for snakebite envenomation treatment in the near future.

## Background

Snakebite is a worldwide medical problem, particularly in tropical or subtropical areas including Taiwan. Globally, an estimation of 125,000 deaths are reported due to snakebites every year [1,2,3,4]. It is believed that the number of cases are underestimated because most snakebites occur in remote or rural areas. Asian *Trimeresurus* snakes are one of the most diverse adaptive groups of venomous pit vipers [5], which include a monophyletic cluster of over 30 species. The members of the *Trimeresurus* family diversify rapidly in ecology, life-evolution, and individual behavior [6]. *Trimeresurus stejnegeri* (TS; formerly *Trimeresurus gramineus*), also known as green bamboo vipers, are best recognized for their remarkable similarity in morphology. 

TS is widely found in southern China, southeastern Asia, and Taiwan, where it is responsible for most snakebites. This species is classified into three significant subspecies, including *T. s. yunnanensis* in India and Myanmar, *T. s. chenbihuii* in China and Myanmar, and *T. s. stejnegeri* in China, Vietnam, and Taiwan [7]. Previous studies indicated that venom proteins of *T. stejnegeri* showed significant geographic variation concerning their morphology, content of mitochondrial DNA and toxic components [6,8,9,10,11,12]. TS proteins exhibit hemotoxic activity and are composed of sophisticated substances with different biological functions, including phospholipase A2 (PLA2), metalloproteinases, hyaluronidases, and thrombin-like serine protease. Such rich mixture leads the victims to hemorrhagic symptoms and even death [13,14,15,16]. Thus, it is highly demanding to develop therapeutic antidotes against specific components in the venom proteins. So far, equine-derived antivenom is the most common antidote available for treating snake envenomation. However, the production of antivenom in horses is expensive, requires labor-intensive fostering and further refinement of IgG antibodies from serum. Besides, repeated administration of equine-derived antivenom often causes severe side effects such as serum sickness or anaphylactic shock responses [3,17]. 

To solve the problems associated with the production and clinical application of equine-derived antibodies, chickens could be an alternative for antibody production, since they are less expensive to nurture and easy to handle [18,19,20]. Large amounts of polyclonal immunoglobulin could be easily extracted and purified from the egg yolk (IgY antibodies) without bleeding [21]. In general, 100-150 mg of IgY antibodies could be obtained, in which approximately 2-10% is specific against the immunized antigen [22]. In addition, the problems associated with the preparation of snake venom proteins could be solved, because only little antigens are necessary to elicit a robust humoral antibody response in chickens, thus making them a perfect alternative model for generating antigen-specific antibodies [23]. Previous studies have reported that IgY antibodies could have neutralizing ability without serious side effects during passive immunization. This advantage could be a shorter and cheaper way for therapeutic applications [24,25]. 

However, it is well known that polyclonal antibodies, including IgY, consist of a group of antibodies with diverse binding activities, resulting in their low specific activity to targeted antigen, and thus limiting their applications for therapeutic or diagnostic purposes. The cross-reactivity inherited in polyclonal antibodies often causes unwanted harmful effects. Therefore, the quality of polyclonal antibodies varies significantly depending on the make-up of the antigens, the production methods, and the animal hosts [26]. By contrast, monoclonal antibodies recognize one particular epitope providing them with high antigen-specificity and low cross-reactivity. This property has the benefits of being widely applied in basic and clinical researches [27,28]. Although the binding efficacy of single monoclonal antibody might be lower than polyclonal antibodies when used in neutralizing snake venom proteins, a combination of various monoclonal antibodies has been greatly shown to reduce symptoms, increase the survival time, and even prevent death [29]. Monoclonal antibodies were also used as a diagnostic agent to ascertain the type of snake envenomation on victims [30]. A hybridoma is the first technology used to produce specific monoclonal antibodies, which requires a tedious and expensive process [31]. Nowadays, phage display technology has become a more convenient, rapid, and inexpensive way to generate specific antibodies from constructed antibody libraries [32]. Most noticeably, it is particularly feasible to create antibodies in the form of a single-chain variable fragment (scFv) or antigen-binding fragment (Fab) using this technology. A monoclonal scFv antibody composed of a light chain variable (V_L_) and a heavy chain variable region (V_H_) joined by a short peptide linker often retains the high antigen-binding affinity of the parental IgG [33]. 

The studies above indicated that a more convenient and cost-effective host is needed to replace the traditional way of antivenom generation from horses. Moreover, monoclonal antibodies with better specificity offer more precise diagnosis of snakebites for determining proper regimens within short time. In this context, chickens were regarded as appropriate and widely handy hosts for the generation of polyclonal and monoclonal antibodies in various fields [34,35]. In this study, polyclonal IgY antibodies were significantly elicited in chickens immunized with TS venom proteins. Later, monoclonal scFv antibodies were produced by phage display technology. The binding activities of IgY and scFv antibodies were further analyzed on immunoblots and enzyme-linked immunosorbent assay (ELISA). The data concluded that the obtained scFv antibodies specifically recognize TS proteins with some cross-reactivity with TM venom proteins. Noticeably, IgY antibodies provided full protection to mice challenged with a lethal dose of TS proteins, In contrast, the mixture of scFv antibodies decreased the mortality and extended the survival time of mice. Thus, we concluded that chicken-derived IgY and scFv antibodies have significant values in the development of diagnostic and therapeutic agents for snake envenomation after advanced clinical trials. 

## Methods

### Animal models

Animals used in the experiments were approved by the Institutional Animal Care and Use Committee at the Taipei Medical University. Therefore, White Leghorn (*Gallus domesticus*) hens, aging 6 months, and ICR mice, weighing 12-14 g, were purchased from the National Laboratory Animal Center, Taiwan. Animals were kept in the animal core facility of the Taipei Medical University (Ethical approval code: LAC-2017-0253; valid on 2017/11/15).

### Hen immunization

We dissolved TS venom proteins in phosphate-buffered saline (PBS), which were generously provided by Taiwan Centers for Disease Control (Taiwan CDC). We mixed 0.125% glutaraldehyde (GA; Sigma, USA) with TS venom proteins to attenuate the hemotoxic activity in the dark at room temperature (RT) for 1 h [ 36,37 ]. For the first immunization, 100 μg of TS proteins in 250 μL PBS were mixed with an equal volume of complete Freund’s adjuvant to inoculate distinct regions of chicken legs intramuscularly. Following immunizations, 80 μg of TS proteins in 250 μL PBS mixed with incomplete Freund’s adjuvant vaccinated at weekly intervals. Polyclonal IgY antibodies in eggs from pre-immunization and the 5^th^ immunization were purified using dextran sulfate/calcium chloride for lipoproteins precipitation and sodium sulfate for IgY antibodies precipitation by centrifugation as described previously [38,39 ].

### Construction of antibody libraries

Recombinant phages displaying antibodies were generated as previously reported 40,41. In short, the spleens of hens after one week of 5^th^ immunization were removed and homogenized in Trizol solution (Invitrogen, Carlsbad, CA, USA) to extract total RNAs according to the manufacturer’s instruction. The cDNA synthesis was performed in a 50 μL reaction containing reverse transcriptase, whose products were used as templates to amplify V_L_ and V_H_ regions of IgY immunoglobulins using polymerase chain reaction (PCR). The PCR-amplified V_L_ and V_H_ fragments joined by a peptide linker containing amino acids GQSSRSS or GQSSRSSSGGGSPGGGGS to form a combinatorial scFv antibody gene fragments by overlapping PCR. The scFv fragments were digested with SfiI (New England Biolabs, Ipswich, CA, USA) and ligated into the pComb3X vector, whose products were electroporated into *Escherichia coli* (*E. coli*) ER2738 (*SupE*) host (MicroPulser, Bio-Rad, Hercules, CA, USA). The dimension of the constructed antibody library was measured using an aliquot of the transformed *E. coli* cells on LB agar plates containing 50 μg/mL of Ampicillin (Amp). The remaining *E. coli* culture was infected with 10^12^ plaque-forming unit (pfu) of VCS-M13 phages and incubated at 37 ^0^C overnight. After centrifugation at 3,000 rpm for 20 minutes, the recombinant M13 phages in the supernatant were precipitated on ice for 30 minutes after the addition of 4% polyethylene glycol (PEG) 8000 and 3% NaCl, were then re-suspended in 1× PBS containing 1% bovine serum albumin (BSA) and 20% glycerol. Finally, the recovered M13 phages were titrated and used for subsequent biopanning.

### Biopanning for phages displaying anti-TS scFv antibodies

Biopanning were carried out on ELISA microplates. Briefly, TS venom proteins (10 μg/mL) were coated on wells at 4 °C overnight. The wells were blocked with 3% BSA at 37 °C for 1 h. A total of 10^11^-10^12^ pfu recombinant M13 phages were added and incubated at 37 °C for 2 h. Non-specific phages were removed using PBST (1× PBS containing 0.05% Tween 20) and specific phages displaying anti-TS scFv were washed down using 0.1 M glycine-HCl (pH 2.2) by robust pipetting. After adding 2 M Tris base buffer, the specific phages were amplified by infecting ER2738 *E. coli* cells at 37 °C for overnight, which were then collected, precipitated and re-suspended in 1× PBS with 1% BSA and 20% glycerol for the next round of biopanning. The titers of eluted phages and the amplified phages were determined on LB agar plates using the plaque-forming assay.

### Expression and purification of *E. coli*-derived scFv antibodies

Total phagemid DNAs were purified from ER2738 cells after the 4^th^ biopanning and transformed into non-suppressive TOP10F' *E. coli* cells. Bacterial clones were randomly selected and cultured in a super broth medium containing 1 mM MgCl_2_ and 50 μg/mL of Ampicillin at 37 °C until the optical density reached 0.8. The expression of scFv antibodies was induced by the addition of 1 mM isopropyl-β-D-thiogalactopyranoside (IPTG) at 30 to 37 °C for overnight. The bacterial cells collected by centrifugation at 3,000 rpm were lysed in histidine (His) binding buffer (20 mM sodium phosphate, 0.5 M NaCl, 20 mM imidazole, pH 7.4) by sonication. The cell debris removed by centrifugation and the supernatant was mixed with Ni^2+^ Sepharose (GE Healthcare Biosciences AB, Uppsala, Sweden) purified His-tagged scFv antibodies based on the manufacturer’s instructions. The purified recombinant scFv antibodies were dialyzed in 1× PBS at 4 ^0^C overnight and then concentrated using Amicon Ultra-4 Centrifugal Filter Units (Merck Millipore, Darmstadt, Germany). 

### Western blotting

TS venom proteins separated on 15% SDS-PAGE immobilized on polyvinylidene fluoride (PVDF) membranes, incubated with 5% skim milk in 1× PBS at 25 °C for 1 h. The proteins were incubated with horse-derived antivenom in 1:1,000 dilution at 25 °C for 1 h , washed three times with PBST and then incubated with horseradish peroxidase (HRP)-conjugated goat anti-horse Fab (Jackson ImmunoResearch, West Grove, PA, USA) as the secondary antibody. After three washes, the binding signal of anti-TS antibodies was detected by adding diaminobenzidine (DAB) substrate. Following a similar procedure, the anti-TS binding activity of IgY antibodies purified from the egg yolk of hens from pre- and post-immunization were tested using HRP-conjugated donkey anti-chicken IgY (Jackson ImmunoResearch, West Grove, PA, USA) as the secondary antibody. 

To detect the binding specificity of anti-TS scFv antibodies, the PVDF membranes immobilized with venom proteins (10 μg/well) of *Deinagkistrodon acutus* (DA), *Bungarus multicinctus* (BM), *Trimeresurus mucrosquamatus* (TM), *Naja naja atra* (NNA), *Daboia russelii formosensis* (DRF) from Taiwan CDC, and TS were incubated with each purified scFv antibody expressed in *E. coli*. The bound anti-TS scFv antibodies were detected by adding a goat anti-chicken light chain (Bethyl, Laboratories, Montgomery, TX, USA); as the secondary antibody, followed by HRP-conjugated donkey anti-goat IgG (Jackson ImmunoResearch, West Grove, PA, USA) as the third antibody. Steps for blocking, washing, incubation, and color development were carried out as stated above.

### Enzyme-linked immunosorbent assay (ELISA) and competitive ELISA

TS venom proteins (0.5 μg/well) and BSA (0.5 μg/well) dissolved in PBS were coated on ELISA wells at 37 °C for 1 h, followed by blocking with 1× PBS (5% skim milk) for one additional hour. IgY antibodies purified from pre-immunized and immunized chickens, were 2× serially diluted (500x to 256,000x), and added into the wells for 1 h. After washing vigorously with PBST, HRP-conjugated donkey anti-chicken IgY were applied and incubated at 37 °C for 1 h. After washing the above, the binding signals were detected using 3, 3′, 5, 5′-tetramethylbenzidine (TMB) substrate. The reactions were terminated, and the optical density was measured at 450 nm. For phage-based ELISA, amplified phages (10^11^-10^12^ pfu) after each biopanning was incubated with the immobilized TS venom proteins, followed by adding HRP-conjugated mouse anti-M13 antibodies (GE Healthcare Bio-Sciences, Marlborough, MA, USA). Similarly, the screened scFv antibodies (5 μg/mL) were incubated with the venom proteins of DA, BM, TS, TM, NNA, and DRF. After that, goat anti-chicken light chain IgG was added, followed by adding HRP-conjugated donkey anti-goat IgG antibodies for detection. 

For competitive ELISA, TS venom proteins (400 μg/mL to 0.40 μg/mL) were pre-incubated with an equal volume of scFv antibodies (10 μg/mL) at 25 °C for 1 h. The mixtures were then incubated with the immobilized TS proteins in wells at 37 °C for 1 h. The blocking, washing, incubation, and color development were carried out following the steps described above. All the results are shown as the mean ± SD from at least two independent experiments.

### Sequence analysis of *E. coli*-derived scFv antibodies

The nucleotide sequences of V_L_ and V_H_ genes of randomly selected anti-TS scFv clones were determined with ompseq primer (5′-AAGACAGCTATCGCGATTGCAGTG-3′) using the ABI 3730 XL auto sequencer (Applied Biosystems, Foster City, USA) [40]. The amino acid sequences of scFv antibodies were deduced to reveal the locations of frameworks (FRs) and complementarity determining regions (CDRs), following the alignment with that of avian immunoglobulin germline gene using the BioEdit alignment program [42].

### Neutralization assay of *E. coli*-derived scFv antibodies

The neutralizing activity of anti-TS scFv antibodies was tested following the WHO and Taiwan CDC protocol [2,37]. Instead of using LD50 according to the WHO protocol, we chose to use absolute lethal dose (LD_100_), lowest dose causing 100% of mice death, as a more precise way by referring the Taiwan CDC regulation of horse-derived anti-venom neutralizing assay. By the reference from Taiwan CDC, the average of 1×LD_100_ for TS venom proteins was 22 μg for 12-14 g mice. We used 0.5× (11 μg), 1× (22 μg), 1.5× (33 μg) and 2× (44 μg) for testing LD_100_. A volume of 200 μL of 1× PBS containing TS venom proteins (11, 22, 33, or 44 μg) was incubated at 37 °C for 1 h and intraperitoneally injected into a group of 9 ICR mice to determine the absolute lethal dose (LD_100_). PBS only was used as a control. Thereafter, yolk IgY antibodies (4 mg/each) from pre-immunized chickens or 5^th^-immunized chickens, horse-derived IgG antivenom (4 mg) (branch no: 60-06-0010; manufacturer: Taiwan CDC; expiry date: 2020/02/01) or a mixed scFv antibodies (4 mg) were individually incubated with TS venom proteins (33 μg) in 200 μL of 1× PBS at 37 °C for 1 h. The final mixtures were injected into the mice intraperitoneally. The mice were monitored continuously for 36 h.

### Statistical analyses

The *in vivo* mice model for testing the neutralizing activity of IgY and scFv antibodies were analyzed via the Gehan-Breslow-Wilcoxon program using GraphPad Prism 6 software (La Jolla, CA, USA). *P* values less than 0.05 were regarded to be statistically significant.

## Results

### Characterization of anti-TS IgY antibodies

**Figure 1.**
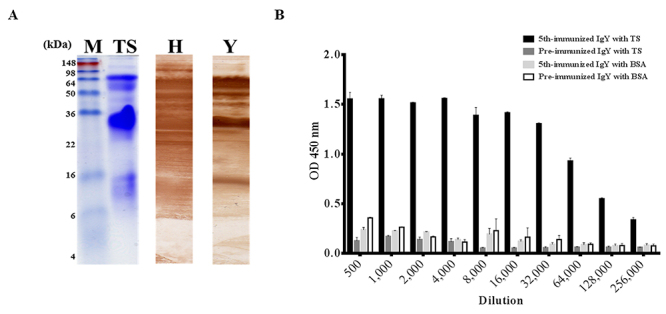
Analysis of hen-derived anti-TS IgY in the yolk. **(A)** The TS venom proteins were checked by 15% SDS-PAGE stained with Coomassie blue dye (lane TS). After immobilized onto PVDF blots, the TS proteins were detected using equine-derived antivenom (lane H) or hen-derived IgY antibodies after 5^th^ immunization (lane Y) as reported in the text. **(B)** Purified IgY from pre-immunized hens (pre-immunized IgY) or hens immunized 5 times (5^th^-immunized IgY) 2-fold diluted serially (500× to 256,000×) was utilized to examine their binding affinity to TS venom proteins or BSA on ELISA plates, respectively. Lane M contains the pre-stained protein markers.

The components of TS venom proteins were visualized on SDS-PAGE (Fig. 1A, lane TS). Of note, two vital proteins with molecular weights of approximately 70 kDa and 34 kDa were observed. Horse-derived IgG antivenom (lane H) and purified IgY (lane Y) antibodies from 5^th^-immunized chickens exhibited different binding patterns on immunoblots. Interestingly, in contrast to IgG, IgY antibodies primarily recognized several proteins of 70-55 kDa, 34 kDa, and 15 kDa. The humoral antibody response was further monitored using ELISA. The results showed that polyclonal IgY antibodies (32,000× dilution) reacted strongly to TS proteins (ODs > 1.0) but not to BSA (Fig. 1B).

### Construction of scFv-displaying phage libraries

Total RNAs were extracted from the spleens of immunized hens to synthesize the cDNA copies. Two successive PCR was performed to amplify the full-length scFv genes. The V_H_ (400 bps) and the V_L_ (350 bps) fragments were first amplified, which products were linked and extended by an overlapping PCR to form scFv fragments (750 bps) containing either a short or long linker (scFv-S or scFv-L), followed by cloning and expression in *E. coli*. The phage clones in two antibody libraries were calculated to be 2.4 × 10^7^ and 6.8 × 10^7^, respectively. After infecting M13 helper phage, the recombinant scFv-displaying phages were used for biopanning. 

### Selection of specific scFv antibodies

**Figure 2.**
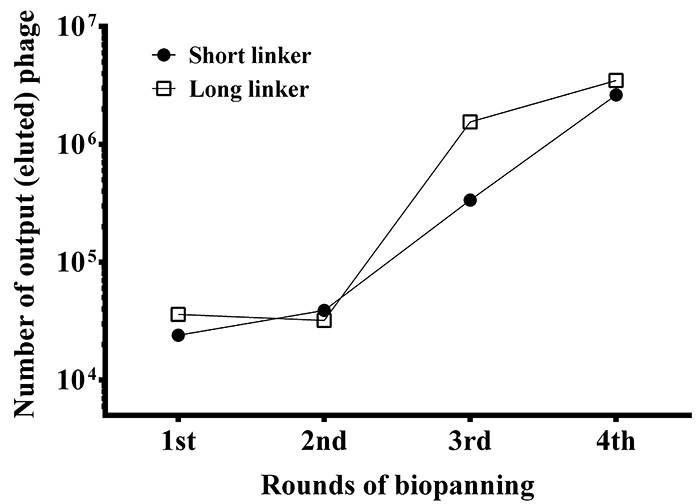
Analysis of phage titers throughout the biopanning steps. The recombinant phages of two antibody libraries were eluted after each biopanning to infect the *E. coli* host. Their titers were determined using a colony formation assay.

Four rounds of biopanning were performed to select anti-TS scFv antibodies. The titers of eluted phages were determined after each biopanning (Fig. 2). The eluted phage titers of scFv-S and scFv-L libraries were estimated to be 2 × 10^4^ and 4 × 10^4^ colony-forming unit (CFU) in the first biopanning, respectively, remained in the same level in the second round and increased steadily thereafter. A dramatic increase in titers was observed in the next 2 rounds of biopanning. Similar patterns were also found in previous studies on the generation of anti-DRF and anti-NAA scFv antibodies [43,44]. The results indicated that scFv-displaying phages with anti-TS activities were significantly enhanced throughout the biopanning procedures.

### Characterization of scFv antibodies

**Figure 3.**
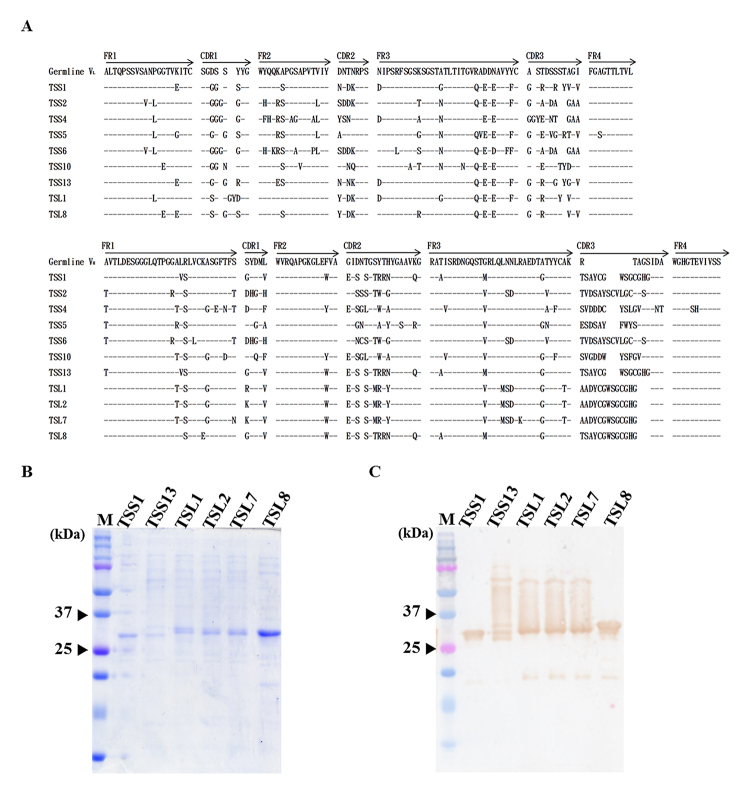
Analysis of sequence homology of V_L_ and V_H_ genes and purified anti-TS scFv antibodies. **(A)** Thirty scFv clones (15 containing short linker and 15 long linkers) were chosen after 4^th^ biopanning and determined their nucleotide sequences. The deduced amino acid sequences using the BioEdit program were compared to that of the chicken’s germline gene. Sequence gaps were launched with blank spaces to optimize the alignment. The dashes (-) represent the same amino acid sequences. Arrows on top of amino acid sequences of germline represent the domains of framework regions (FRs) and complementarity-determining regions (CDRs). **(B)** After adding IPTG for induction, His-fused scFvs (lanes TSS1 to TSL8) with binding activities to TS proteins using Ni^2+^ Sepharose were purified and analyzed their purity on SDS-PAGE stained with Coomassie blue dye. **(C)** Their identities were further verified using goat anti-chicken light chain antibody, followed by HRP-tagged donkey anti-goat IgG on Western blots. Approximately 0.1 μg of each scFv antibody was used for analysis.

Thirteen clones from scFv-S or scFv-L libraries (26 clones in total) were randomly chosen to analyze their scFv antibody expression and confirm the His-fused scFv antibodies using mouse anti-His antibody on western blot. (data not shown). The nucleotide sequences of V_L_ and V_H_ genes of scFv positive clones were determined and aligned with the chicken immunoglobulin germline gene. The results showed that seven distinct groups of scFv antibodies enriched from scFv-S library were identified and represented as TSS1 (3/13; 23%), TSS2 (1/13; 7.7%), TSS4 (1/13; 7.7%), TSS5 (3/13; 23%), TSS6 (1/13; 7.7%), TSS10 (3/13; 23%), and TSS13 (1/13; 7.7%). Similarly, four groups of scFv antibodies from scFv-L library were identified and represented as TSL1 (7/13; 54%), TSL2 (4/15; 31%), TSL7 (1/15; 7.5%) and TSL8 (1/15; 7.5%) (Table 1). The amino acid sequences of V_L_ and V_H_ regions were deduced and aligned with those of chicken germline, as shown in Figure 3A. Significant variations were observed in the complementarity-determining regions (CDRs), particularly the CDR3s in V_L_ and V_H_ genes (36%~90% and 45~81% mutation rates, respectively), as compared with the framework regions (FRs) (Table 2). Intriguingly, TSL1, TSL2, and TSL7 used the identical V_L_ genes paired with divergent V_H_ genes to construct functional anti-TS scFv antibodies. The biological significance of the same V_L_ gene usage is currently not known. The results together implied that these selected scFv antibodies were produced from stimulated B cells but not directly from naive B cells, suggesting that a strong antigen-driven humoral antibody response was elicited in chickens after immunization. 

**Table 1:** Classification of anti-TS scFv clones according to the identity of V_L_ and V_H_ regions.

Groups	Short linker			Long linker		
	V_L_	V_H_	Percentage	V_L_	V_H_	Percentage
Group 1	1, 3, 8	1, 3, 8	23%	1, 2, 3, 4, 5, 6, 7, 9, 10, 11, 12, 13	1, 4, 6, 9, 11, 12, 13	54%
Group 2	2	2	7.7%		2, 3, 5, 10	31%
Group 3	4	4	7.7%		7	7.5%
Group 4	5, 7, 9	5, 7, 9	23%	8	8	7.5%
Group 5	6	6	7.7%			
Group 6	10, 11, 12	10, 11, 12	23%			
Group 7	13	13	7.7%			

**Table 2:** Amino acid mutation rates of single-chain variable fragment (scFv) clones

Region	CDR1	CDR2	CDR3	Total CDRs	FR1	FR2	FR3	FR4	Total FRs
V_L_	30~50%	14~57%	36~90%	35~64%	5~10%	0~50%	13%~25%	0~10%	9%~22%
V_H_	40~80%	35~50%	63~81%	45~62%	7~23%	0~7%	9%~22%	0~18%	7%~16%

CDRs: complementarity domain regions; FRs: framework regions; V_L_: variable region in light chain; V_H_: variable region in heavy chain.

After IPTG induction, these scFv antibodies in the cell lysates were examined for binding activities on ELISA. Of which, 6 scFv clones (TSS1, TSS13, TSL1, TSL2, TSL7, and TSL8) showed strong binding signal to TS proteins, but the other 5 (TSS2, TSS4, TSS5, TSS6, and TSS10) did not (data not shown). These binding-positive scFv antibodies were purified and analyzed on SDS-PAGE and immunoblots. A major band with 30 kDa in molecular weight was visualized, implying that these recombinant scFv antibodies were appropriately expressed (Fig. 3B). Anti-chicken light chain antibodies further validated the identity of the expressed scFv antibodies (Fig. 3C). However, other than the major band, an additional protein with larger molecular weights was recognized in the preparation of purified TSS13, TSL1, TSL2, and TSL7 antibodies on western blots. These proteins’ identities need further characterization but they were reasoned to be the dimeric form of scFv antibodies. The problem may be partially answered using urea-containing elution buffer during antibody purification. 

### Binding specificity of selected scFv antibodies

**Figure 4.**
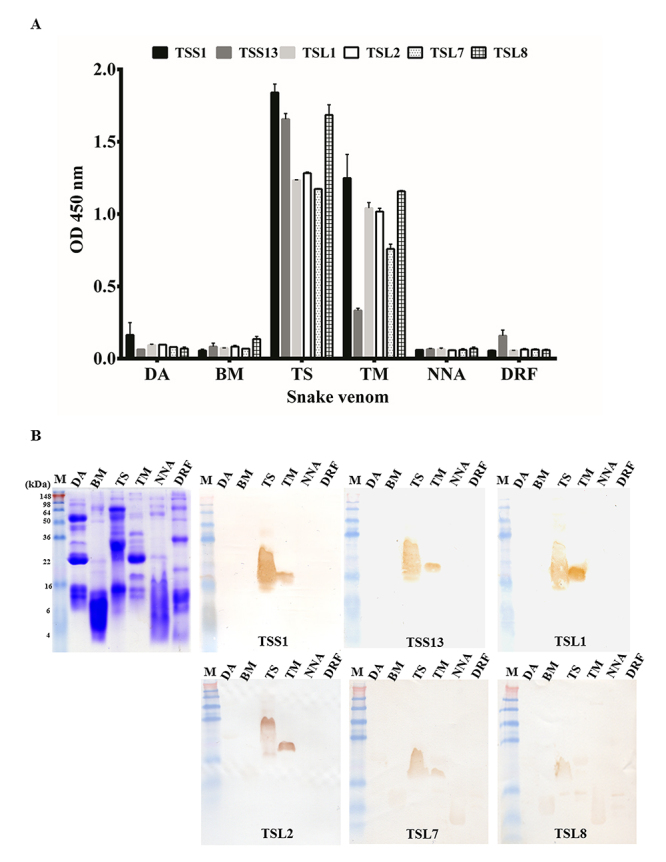
Binding analysis of anti-TS scFvs to various venom proteins. **(A)** Venom proteins collected from snakes DA, BM, TS, TM, NNA, and DRF were coated on ELISA wells and incubated with the individual, purified scFvs (5 μg/mL). **(B)** Their binding specificity against particular components in the venom proteins was further examined and visualized on Western blots. Each venom protein was loaded 10 μg/well.

Venom proteins of six venomous snakes (DA, BM, TS, TM, NNA, and DRF) were coated on ELISA wells or PVDF papers for binding analysis. The ELISA data showed that purified scFv antibodies exhibited diverse degrees of binding activities (ODs > 1.1) to TS proteins (Fig. 4A). Interestingly, they also showed cross-reactivity to TM venom proteins in fewer levels (Fig. 4A). Their binding specificity was further verified on immunoblots (Fig. 4B). They recognized a smeared TS protein of 35-20 kDa and a distinct TM protein of 20 kDa with various levels of binding activities. No significant cross-reactivity to the venom proteins DA, BM, NNA, and DRF were observed on either ELISA or immunoblots. In short, these data indicated that this anti-TS scFv might recognize the highly homologous proteins in hemotoxic venomous *Trimeresurus* snake species. However, we presently have no evidence to support this presumption.

### Competitive ELISA

**Figure 5.**
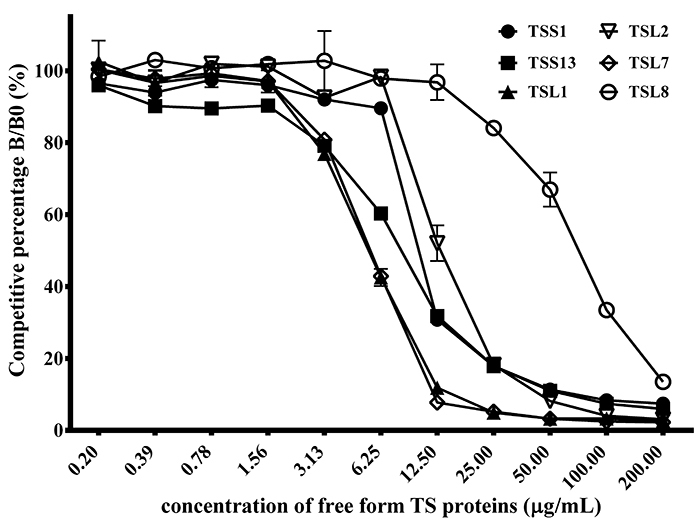
Binding analysis of anti-TS scFvs using competitive ELISA. Individual scFv was purified, incubated with several amounts of soluble TS venom proteins, and added to the ELISA wells coated with TS venom proteins as described in the text. The inhibitory percentage was shown as B/B0, representing the number of bound scFvs in the presence or absence of soluble TS venom proteins, respectively. ELISA data were the means of duplicated experiments.

Competitive ELISAs were carried out further verify the binding activities of anti-TS scFv antibodies. Individual anti-TS scFv antibody was first incubated with free TS venom proteins, which was later added to the ELISA plates coated with TS proteins. Optical density in the absence of free TS proteins was taken as 100% against those in the presence of various concentrations of free TS proteins to estimate the percentage of inhibitory effects. The data showed that their binding activities to TS proteins were suppressed in dose-dependent manners (Fig. 5). Only 6.25 μg/mL of free TS proteins were needed to achieve more than 50% suppression on binding activities of TSL1 (57%) and TSL7 (57%). Likewise, 12.5, 12.5, 25, and 100 μg/mL of TS proteins were needed to achieve the same inhibitory effects on binding activities of TSS1 (69%), TSS13 (68%), TSL2 (82%) and TSL8 (67%). In each scFv group, we selected three TS proteins concentrations whose suppression percentage was the closest to 50% suppression to calculate the linear regression and the TS proteins concentration at 50% suppression (Additional file 1). The amount of the recognized proteins was predicted by ImageJ software. Thus, the dissociation constant (*K*_*d*_ ) of these six scFv antibodies was 13.84 ± 8.383 × 10^-8^, 8.48 ± 5.127 × 10^-8^, 6.55 ± 3.967 × 10^-8^, 15.26 ± 9.256 × 10^-8^, 6.66 ± 4.031 × 10^-8^ and 83.25 ± 50.558 × 10^-8^ M as calculated by the Klotz plot method (Table 3) [45]. These results demonstrated these scFv antibodies possessed significant and yet similar anti-TS activities. 

**Table 3.** Calculated dissociation constant (*Kd*) values of anti-TS single-chain variable fragments (scFv) antibodies.

Clone	Linear regression	Inhibition of 50% binding of recognized proteins (μg/mL)	***Kd* values** **(M)**
TSSS1	y = -3.4249x + 96.032	4.03 ± 3.084	13.84 ± 8.383 × 10^-8^
TSSS13	y = -2.1013x + 67.305	2.47 ± 2.331	8.48 ± 5.127 × 10^-8^
TSL1	y = -6.6462x + 92.247	1.91 ± 1.799	6.55 ± 3.967 × 10^-8^
TSL2	y = -4.0496x + 115.23	4.44 ± 4.186	15.26 ± 9.256 × 10^-8^
TSL7	y = -7.4837x + 98.406	1.94 ± 1.830	6.66 ± 4.031 × 10^-8^
TSL8	y = -0.334x + 76.949	24.21 ± 22.826	83.25 ± 50.558 × 10^-8^

### *In vivo* neutralization assay

**Figure 6.**
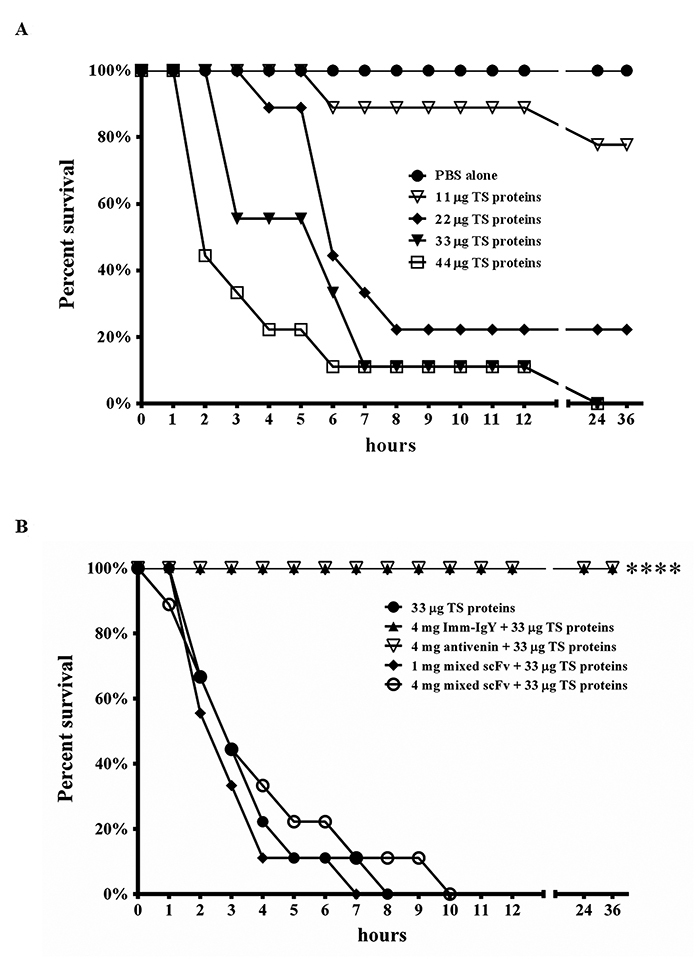
Neutralization analysis of anti-TS scFvs in mice. **(A)** Clusters of 9 ICR mice were challenged with several amounts of TS protein proteins (11, 22, 33, and 44 μg) in peritoneal space to establish the 1×LD_100_. **(B)** Polyclonal IgY antibodies from pre-immunized (Pre-IgY) or immunized (Imm-IgY) hens, equine-derived antivenom (4 mg), or a combination of six anti-TS scFvs (1 and 4 mg) were individually incubated with TS venom proteins at 37 °C for 1 h. These preparations were injected into mice, which were recorded hourly for 36 h.

To determine the LD_100_, we intraperitoneally injected the mice with 11, 22, 33, or 44 μg of TS venom proteins (Fig. 6A). Administration of 11 μg of TS proteins led to the death of 1 mouse within 6 h and 1 within 24 h; 7 mice survived without obvious abnormality. Administration of 22 μg of TS proteins led to the death of 1 mouse within 4 h, 4 within 6 h, 1 within 7 h and 1 within 8 h, 2 survived normally. By contrast, all the mice administrated with either 33 μg or 44 μg of TS proteins died within 24 h, while 100% survival rates were recorded in PBS-treated mice. Thus, 33 μg of TS proteins were taken as 1×LD_100_ for neutralization studies. In contrast, 4 mg of anti-TS IgY from 5^th^-immunized chickens or horse-derived antivenom provided full protection to envenomed mice. We further analyzed the inhibitory effect of 1 mg and 4 mg of combined anti-TS scFv antibodies on mice. All mice with 1 mg treatment died within 7 h while 8 mice with 4 mg treatment killed within 7 h and 1 survived for 10 h. The data suggested that the anti-TS scFv antibodies obtained offer minimal neutralizing activity against the lethal effect of TS venom proteins on mice. 

## Discussion

The supply of enough quantity of snake venom proteins is one major limitation for generating a neutralization antibody. Protocols from the World Health Organization (WHO) [2,3,37] suggested that horses be administrated with 1-4 mg of snake venom proteins in the first immunization and 5-10 mg in following immunizations in a 2-week duration until the specific antibody titers reach the plateau. It means that horses may require approximately 20-45 mg for 5 times of immunization in 2 months. In this study, hens were first administrated with 100 μg of TS venom proteins and subsequently 80 μg in a 1-week duration for 4 times. A total of 420 μg of venom proteins were required to elicit significant anti-TS antibody response for 5 times of immunization in 5 weeks. As shown in Figure 6B, a small dosage of TS venom proteins were difficult to collect, was sufficient to elicit neutralizing antibodies in hens. In addition, according to WHO Guidelines, the collection and refinement of horse serum are more difficult for the production of antivenom in various seasons and areas [37]. These results suggested that hens are a more cost-effective host for antibody production. Other studies also concluded that the cost of antibody production was 30%-40% cheaper in hens than horses, especially in developing countries [46]. Numerous studies on IgY used for immunotherapy in clinical and experimental treatments have been reported [24,25]. We were aware that more detailed studies should be carried out to illustrate the difference between IgY and IgG antibodies before the chicken-derived IgY antibodies are released for clinical use. We still believed that chicken-derived IgY antibodies could be potentially used for therapeutic applications in the future.

Phage display technology was developed by Smith [47] and proven to be a valuable platform for studying the mechanisms of protein-protein interaction in various researches, including drug discovery and antibody generation [48]. The chicken animal model with phage display technology offers a more affordable and available way for generating of monoclonal antibodies and could be considered a potential source of antibodies for clinical applications [32,49]. One advantage of producing antibodies in hens using phage display technology is their relatively simple immunoglobulin repertoire, making it easier to construct antibody libraries from a solitary association of V_L_ and V_H_ genes [50]. In this study, we established two scFv antibody libraries with 2.4 × 10^7^ and 6.8 × 10^7^ phage clones from hens administered with TS venom proteins. Our previous studies have shown that the size of libraries was sufficient for the generation of specific antibodies [43,44,51,52,53,54]. Despite generating from naïve antibody libraries, which avoid animal immunization, it requires sizable libraries and extensive biopanning steps for the generation of specific antibodies [55]. In contrast, hyper-immunized animals like chickens in our studies showed more feasible and timesaving in the production of specific antibodies. 

As noted above, the analysis of deduced amino acids of anti-TS scFv randomly chosen after the final biopanning clearly showed major variations in V_L_ and V_H_ gene usage. These were sorted into seven short and four long linker groups, as listed in Figure 3A, and Table 1. However, even though TSS2, TSS4, TSS5, TSS6, and TSS10 were abundantly expressed, they showed no detection of TS venom proteins’ signal to ELISA (data not shown). We thought these five scFvs might have different configurations when expressed on phage and in *E. coli* leading to change in binding activities, or these scFvs were not routed to the periplasm and were not well folded. In contrast, TSS1, TSS13, TSL1, TSL2, TSL7, and TSL8 showed significant binding activities to TS venom proteins on ELISA and immunoblots. Intriguing, they also showed cross-reactivities to TM proteins (Fig. 4A and 4B). Considering that both *Trimeresurus stejnegeri* and *Trimeresurus mucrosquamatus* belong to the family of Viperidae, we deliberated that phospholipase A2, snake venom serine protease (SVSP) and cysteine-rich secretory protein (CRISP) present in both TS and TM venom proteins may be responsible for these observations [56]. It was possible that these screened anti-TS scFv antibodies may recognize antigenic epitope(s) conserved in several TS and TM venom proteins or one protein with different post-translational modifications. However, the underlying mechanism is not precisely known.

The CDR3 fragments in V_H_ genes of IgY generally contained 8 to 32 amino acid residues (mean 16.2 ± 3.2) similar to those of human IgG (5 to 37 amino acids, mean 16.1 ± 4.1). Close to 89% of CDR3 fragments contained 15 to 23 amino acids [42,57]. In contrast, CDR3 of V_H_ of anti-TS scFv in our study contained 16 to 19 amino acids, 82% of which contained 16 amino acids as shown in those of TSS1, TSS4, TSS5, TSS10, TSS13, TSL1, TSL2, TSL7, and TSL8. However, our results did not give any proof of whether the length of CDR3 fragments of V_H_ of anti-TS scFv played an important role in their binding activities, as reported. Additionally, it is well documented that the functional V_H_ or V_L_ genes produced through V-D-J or V-J join and intensive somatic mutation were reciprocally associated with increasing more diversity [58,59]. In this context, high mutation rates in CDR fragments of V_H_ or V_L_ genes of anti-TS scFv were commonly identified as compared with those of the germline gene. Our analysis indicated that the mutation rates in CDR fragments of V_H_ or V_L_ genes extended 45 to 62% and 35 to 64%, respectively (Fig. 3 and Table 2) while the mutation rates in FR fragments of V_H_ or V_L_ genes extended 7 to 16% and 9 to 22%. Noticeably, the mutation rates in CDR3 of the V_H_ genes of all chosen scFv were 63 to 81%. These figures were in accordance with those of previous studies, supporting that high frequency of somatic hyper-mutations happened in the CDR fragments than FR of the rearranged functional antibodies to increase affinity [59,60]. Thus, this anti-TS scFv was generated and chosen as a result of antigen-driven response and B cells’ affinity maturation in the hens administered with venom proteins. However, the random pairing of V_L_ and V_H_ genes often happened in *E. coli* cells, disputing that the anti-TS scFv may not be produced from genuine antigen-stimulated B cells in hens [32[. Such a problem would not be answered until additional experiments are carried out. 

Unexpectedly, the mixed anti-TS scFv antibodies barely provided protective effects on mice, indicating that they had little capacity to neutralize TS proteins’ lethal toxicity. As noted above, more than 30 major proteins and peptides have been identified in TS proteins. In which, the phospholipase A2, snake venom metalloproteinase (SVMP) and snake venom serine protease (SVSPs) are the main components with lethal activity in the members of Viperidae snakes including *T. stejnegeri* [56,61,62,63,64,65,66,67,68]. The molecular weight of metalloproteinase was reported to be around 20-100 kDa, which were not recognized by anti-TS scFv, as shown in Figure 4. The phospholipase A2 enzymes also contain a large number of homologous proteins of approximately 14-18 kDa [66,69]. The SVSPs with the molecular mass of around 17-67 kDa have various sequence homology and glycosylation levels, usually resulting in undifferentiated forms with slightly different molecular weights and isoelectric points [70]. 

Our previous study indicated that the hemolytic activity of SVSP in TM venom proteins was significantly inhibited by scFv antibodies [51]. However, anti-TS scFv antibodies (TSS1, TSL7, and TSL8) obtained in this study exhibited partial inhibitory activities on SVSPs (Additional file 2 and Additional file 3). Knowing that this anti-TS scFv had little protection on mice suggested that they might not bind to SVMP, PLA2 proteins, and SVSP proteins. Additional studies are required to further confirm the exact underline mechanism of the inhibitory effects. Moreover, since the polyclonal anti-TS IgY antibodies provided complete protection on mice (Fig. 6B), it was believed that additional anti-TS scFv antibodies with neutralizing activities could be obtained after the intensive screening. The results lead us to understand that the B cells producing the scFv with neutralization activity exit in the spleen of the immunized chickens. At present, we do not know the exact reason(s) why no scFv with neutralization activity was obtained after the intensive screening. However, the results may be ascribed to the following possible cause(s):

- the immunoglobulin genes encoding the neutralizing scFv were not amplified and cloned out;
- the neutralizing scFv antibodies were not adequately expressed on the surface of M13 phages;
- the neutralizing scFv antibodies were not eluted during biopanning steps;
- the neutralization activities were abolished after elution by strong acid.
- To partially clarify these speculations, we are in the process of:
- constructing additional antibody libraries;
- eluting the potential scFv with neutralizing activity using venom proteins as competitors;
- cloning any residual immunoglobulin genes in the ELISA wells after acid elution.

With all the experimental design and performance, it is believed that additional anti-TS scFv antibodies with neutralizing activities against proteins that provoke hemorrhagic symptoms in TS venom may be identified. 

## Conclusion

We demonstrated that chickens are cost-effective and suitable alternative hosts for the production of antibodies with neutralizing capacity against snake venom proteins. In addition, using phage display technology to produce monoclonal antibodies is more efficient in terms of costs and time. We are hypothesizing that these anti-TS IgY and scFv antibodies together would have great potential for the development of diagnostic kits including treatments for snake envenomation in the near future.

### Abbreviations

BM: *Bungarus multicinctus*; CDC: Centers for Disease Control; CFU: colony-forming unit; DA: *Deinagkistrodon acutus*; DAB: 3, 3′ diaminobenzidine tetrahydrochloride; DRF: *Daboia russelii formosensis*; ELISA: enzyme-linked immunosorbent assay; h: hour; IgY: immunoglobulin Y; NNA: *Naja naja atra*; PBS: phosphate-buffered saline; pfu: plaque forming unit; scFv: single-chain variable fragment; TM: *Trimeresurus mucrosquamatus*; TMB: 3,3'5,5′-Tetramethylbenzidine; TS: *Trimeresurus stejnegeri*; V_H_: heavy chain variable region; V_L_: light chain variable region.

## Supplementary material

The following online material is available for this article:

Additional file 1. Competitive inhibition assay of six representative single chain variable fragment (scFv) antibodies against the TS venom proteins. The amount of bound scFv in the presence of free TS proteins was measured and expressed as a percentage of the binding of scFv in the absence of TS proteins. B and B0 were the amounts of bound scFv in the presence and absence of the inhibitor, respectively. Three concentrations of TS protein as indicated in between dotted lines were used for calculation of linear regression.

Additional file 2. Inhibitory effect of scFv antibodies on hemolytic activity of TS venom proteins on blood agar plate (BAP). Different concentrations of each scFv antibody, mixed scFv, horse antivenom or IgY from immunized chickens were incubated individually with 10 μg of TS proteins at 37 °C for 1 h, dropped on BAP and then incubated at 37 °C for overnight.

Additional file 3. Neutralization efficiency of IgY or scFv antibodies against TS venom proteins on BAP.
