# Supplementary material for: Chicken antibodies against venom proteins of Trimeresurus stejnegeri in Taiwan
Source: J Venom Anim Toxins Incl Trop Dis. 2020 Nov 20;26:e20200056. doi: 10.1590/1678-9199-JVATITD-2020-0056 (PMC7682652; doi:10.1590/1678-9199-JVATITD-2020-0056)

J Venom Anim Toxins incl Trop Dis

https://doi.org/10.1590/1678-9199-JVATITD-2020-0056

Supplementary Material to "Chicken antibodies against venom proteins of *Trimeresurus stejnegeri* in Taiwan"

Additional file 1. Competitive inhibition assay of six representative single-chain variable fragment (scFv) antibodies against the TS venom proteins. The amount of bound scFv in the presence of free TS proteins was measured and expressed as a percentage of the binding of scFv in the absence of TS proteins. B and B0 were the amounts of bound scFv in the presence and absence of the inhibitor, respectively. Three concentrations of TS protein as indicated in between dotted lines were used for calculation of linear regression.

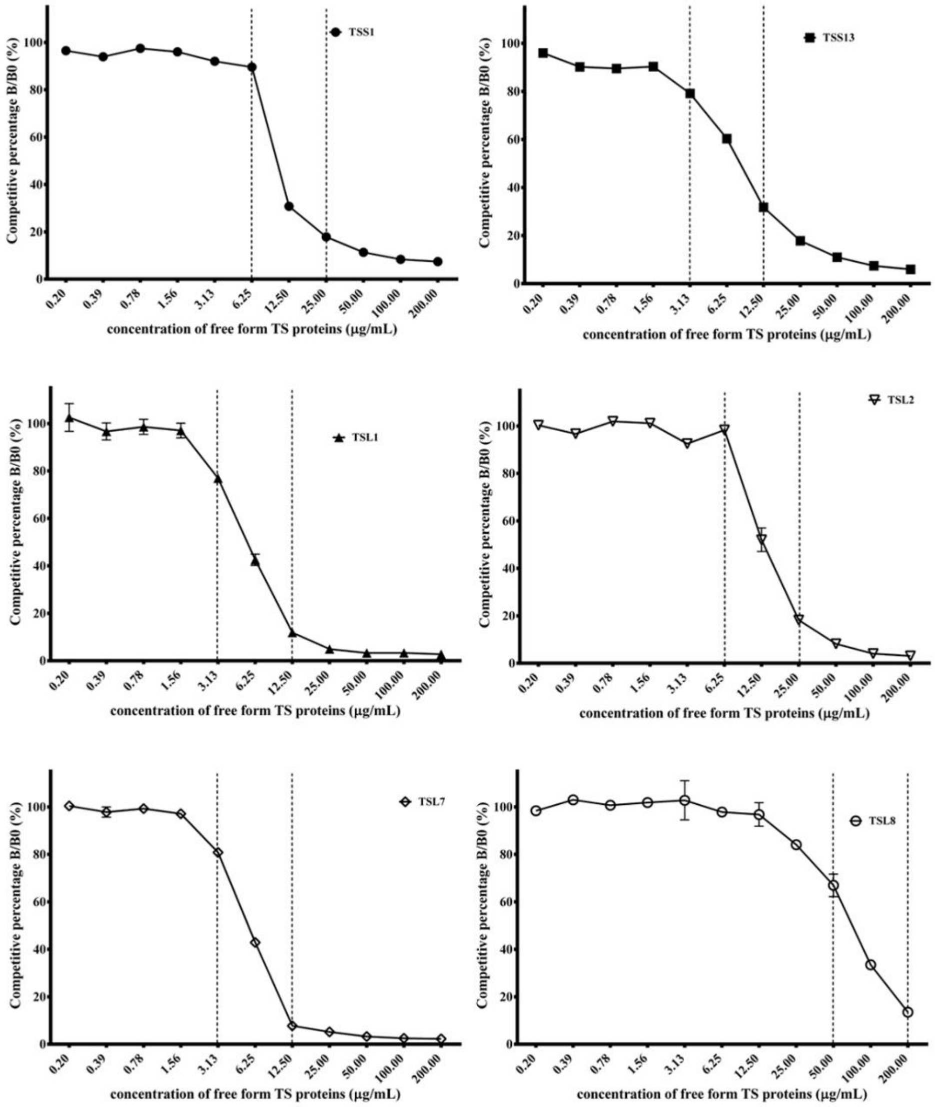

Supplement: Additional file 1. [file 1678-9199-jvatitd-26-e20200056-s1.pdf]
