# Supplementary material for: Chicken antibodies against venom proteins of Trimeresurus stejnegeri in Taiwan
Source: J Venom Anim Toxins Incl Trop Dis. 2020 Nov 20;26:e20200056. doi: 10.1590/1678-9199-JVATITD-2020-0056 (PMC7682652; doi:10.1590/1678-9199-JVATITD-2020-0056)

J Venom Anim Toxins incl Trop Dis

https://doi.org/10.1590/1678-9199-JVATITD-2020-0056

Supplementary Material to "Chicken antibodies against venom proteins of *Trimeresurus stejnegeri* in Taiwan"

Additional file 2. Inhibitory effect of scFv antibodies on hemolytic activity of TS venom proteins on blood agar plate (BAP). Different concentrations of each scFv antibody, mixed scFv, horse antivenom or IgY from immunized chickens were incubated individually with 10 μg of TS proteins at 37 °C for 1 h, dropped on BAP and then incubated at 37 °C for overnight.

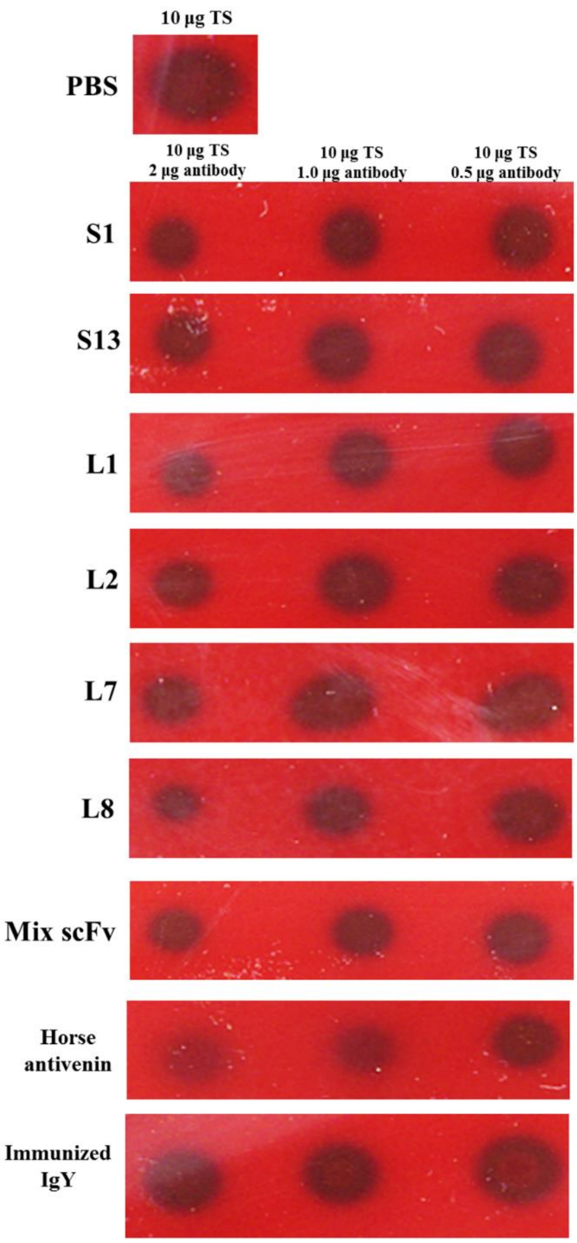

Supplement: Additional file 2. [file 1678-9199-jvatitd-26-e20200056-s2.pdf]
