# Supplementary material for: Chicken antibodies against venom proteins of Trimeresurus stejnegeri in Taiwan
Source: J Venom Anim Toxins Incl Trop Dis. 2020 Nov 20;26:e20200056. doi: 10.1590/1678-9199-JVATITD-2020-0056 (PMC7682652; doi:10.1590/1678-9199-JVATITD-2020-0056)
Supplement: Additional file 3. [file 1678-9199-jvatitd-26-e20200056-s3.pdf]

J Venom Anim Toxins incl Trop Dis

https://doi.org/10.1590/1678-9199-JVATITD-2020-0056

Supplementary Material to "Chicken antibodies against venom proteins of *Trimeresurus stejnegeri* in Taiwan"

Additional file 3. Neutralization efficiency of IgY or scFv antibodies against TS venom proteins on BAP.

Antibody	Amount of antibody		
	2 μg	1 μg	0.5 μg
S1	0.8	0.9	1
S13	0.9	1	1
L1	0.9	1	1
L2	0.8	1	1
L7	0.8	0.9	1
L8	0.7	0.9	1
Mix scFv	0.8	0.9	1
Horse antivenom	0.3	0.4	0.7
Immunized IgY	0.7	0.8	1

* PBS alone: 1 cm
